# Quantifying spatial accessibility to inpatient hospices in Germany

**DOI:** 10.1186/s12913-026-15337-w

**Published:** 2026-08-21

**Authors:** Theresa Petzold, Friedemann Nauck, Christian Banse, Jobst Augustin, Birgit Jaspers, Maximiliane Jansky

**Affiliations:** 1 Department of Palliative Medicine, University Medical Center, Goettingen, Germany; 2 Institute for Health Services Research in Dermatology and Nursing (IVDP), University Medical Center Hamburg-Eppendorf (UKE), Hamburg, Germany

**Keywords:** Health services accessibility, Palliative care, Inpatient hospices, Health geography, Floating catchment area methods, E2SFCA, 2SFCA, GIS, Network analysis

## Abstract

**Background:**

Spatial access is an important indicator to assess quality of healthcare services and healthcare situation, with accessibility and availability as central components. Using inpatient hospices as an example of specialist palliative care in Germany, different indicators are used to measure spatial accessibility, which vary in complexity and considered variables. We compare four widely used methods, identify regional differences, and discuss possible differences in the results caused by methodological characteristics.

**Methods:**

Locations of inpatient hospices were identified using a freely accessible online self-disclosure database, supplemented by lists from regional associations and own research. Spatial access was examined using provider-to-population ratios based on district level, and network-based travel time of 15, 30 and 60 min as used in literature. Further, Two-Step Floating Catchment Area (2SFCA) and Enhanced Two-Step Floating Catchment Area (E2SFCA) were applied using geocoded locations, hospice bed capacities, population data, and road network data.

**Results:**

295 inpatient hospices were identified. 211 of 401 districts have at least one hospice, resulting in an availability of 36.4 beds per 1 million inhabitants nationally. 90.3% of the population can reach a hospice by car within 30 min (network-based travel time). The results of the FCA methods show a heterogeneous pattern in spatial accessibility index (SPAI_i_). Using 2SFCA method results in uniform accessibility within the catchment areas of the hospices. While E2SFCA method with a slow distance decay function generates smoother transition zones within catchment areas, the sharp distance decay function induces higher spatial differentiation, particularly in rural areas, where accessibility declines sharply with distance.

**Conclusions:**

The results indicate that inpatient hospices in Germany are timely accessible via private transport, if available, though rural areas have lower accessibility. However, the chosen method significantly influences the results. Indicators such as provider-to-population ratios and network-based travel time can provide a basic analysis of the care situation due to their ease of interpretation. This study can raise awareness among decision-makers planning new hospices to consider more advanced methods, such as FCA methods, as these take both dimensions of accessibility equally into account.

## Background

Access in a medical context is a complex, multidimensional concept. In addition to the distinction between potential and realized access [1], the dimensions of availability, accessibility, accommodation, affordability and acceptability are most frequently mentioned in this context [2]. Recent concepts add further dimensions such as approachability, appropriateness or awareness [3, 4]. The dimensions of availability and accessibility include among others spatial aspects of potential access and can be summarized under the term ‘spatial accessibility’. This is an important indicator of the quality of healthcare services and the assessment of needs [1, 2, 5, 6]. There is a wide variety of indicators for operationalizing spatial accessibility, which consider either availability or accessibility alone, or both indicators together. The most common indicators are the provider-to-population ratio (PPR), network-based travel time, and the floating catchment area (FCA) method family.

The PPR is the simplest and best-known way of representing the availability of healthcare services. It only describes the ratio between supply and potential demand, without incorporating spatial references such as geographical distances or travel times. The supply is represented by the bed capacity of the healthcare facilities examined. Potential demand is represented by the population or potential patients. The ratio is calculated based on administrative regions (districts, municipalities, etc.) [1, 7].

One possibility for examining accessibility is to perform a network analysis based on travel time. After setting a threshold value, zones of equal accessibility are calculated based on a service location (service areas or polygons) along a road network. Either distance or travel time is taken into account, whereas travel time has a greater influence on patients’ mobility decisions [8, 9]. The service areas are linked to aggregated population data located within the polygons. The potential minimum travel times between the population’s places of residence and the nearest service provider are mapped [10–12].

For a more realistic assessment of the healthcare situation that equally considers both dimensions of spatial accessibility, more advanced methods are needed. Floating catchment area methods have been established in this context for about 20 years. The basic model is the two-step floating catchment area method (2SFCA) [13]. This method uses population figures and the locations of healthcare facilities, including their capacities (usually expressed as the number of beds), to calculate the relationship between supply and demand. Contrary to other methods using fixed administrative boundaries, in 2SFCA catchment areas are flexible (floating catchments), and a maximum travel time is set as a threshold value. Many modifications have been developed based on the 2SFCA method. The most widely used is the enhanced two step floating catchment area method (E2SFCA) by Luo and Qi [14, 15]. Other variations are, for example, the three-step floating catchment area method (3SFCA), the integrated floating catchment method (iFCA) or the modified huff model three-step floating catchment area method (MH3SFCA) [16–18].

Palliative care, as an approach to caring for patients with terminal illnesses, with the aim of improving their quality of life, is explicitly recognized under the human right to health [19–21], and should therefore be available regardless of place of residence, financial means, or social circumstances. There are massive differences in provision of palliative and hospice care services worldwide, with Germany being among the countries with a high standard of care provision [22, 23]. The demand for palliative and hospice care in Germany is expected to increase [24, 25]. Due to ongoing medical advances and the wider range of medical treatment options available, life expectancy has increased and symptom patterns increase in complexity due to multimorbidity [26, 27]. Furthermore, the incidence of both oncological and non-oncological incurable diseases is rising [28]. In Germany, there are numerous outpatient and inpatient, non-specialist as well as specialist palliative care services [29]. Non-specialist palliative care services include basic care provided through general practitioners, ambulant nursing services, nursing homes, and general hospital units, with the involvement of volunteer hospice services in less complex cases. In cases where the patient’s situation is highly complex, treatment is provided by specialist palliative care services through inpatient palliative care units (PCU), hospital palliative care support teams, home palliative care teams (PCT), day hospices and inpatient hospices. In Germany, hospices are inpatient facilities that provide support for the seriously ill with a life expectancy of days, weeks, or a few months if treatment in a hospital is not necessary and care at home or in a nursing facility is not possible or desired. Care is provided by multi-professional teams of nurses, psychosocial and spiritual professionals and volunteers. General practitioners or physicians from PCT provide medical care to patients [24, 29, 30].

Compared to other medical care locations, spatial accessibility to palliative care services in general, and inpatient hospices in particular, has a number of special conditions. For patients, access is mostly only relevant once, as in most cases the hospice will be the place of death. It includes the journey from the patient’s place of residence or from a previous hospital stay, e.g., in a PCU, to the hospice. Transport is usually provided by ambulance or emergency vehicles. Since patients at the end of life often suffer from severe symptoms, long travel times can reduce patient comfort by exacerbating their symptoms, complicate or even prevent transfer to a hospice, and occupy medical transport resources that may be needed for acute and emergency care during that time. Spatial accessibility is particularly relevant for relatives who wish to visit patients at the end of life. Long travel times can be perceived as stressful by patients and their relatives and friends [31, 32] and limit visiting opportunities [33].

In Germany, the first palliative care unit opened in Cologne in 1983 and the first hospice was established in Aachen in 1986. The number of outpatient and inpatient, as well as general and specialist palliative and hospice care services, has grown steadily since that time [34–36]. The German Hospice and Palliative Care Association (DHPV), as the official governing body for hospice and palliative care in Germany, currently reports a total of 350 palliative care units and 260 inpatient hospices [37]. However, little research has been done on the current state of geographical access to inpatient hospices throughout Germany. Previous studies have focused on individual federal states or care services [38–44] or examine either availability or accessibility as dimensions of spatial access, mostly [45–47]. An earlier study conducted in Germany used euclidean distance as an indicator of accessibility [48]. Internationally, studies have been conducted in the United Kingdom [49, 50], Canada [51], Ireland, Spain, and Switzerland [52]. No study conducted in Germany has examined spatial accessibility to inpatient hospices beyond the application of simple availability and PPR. FCA methods have rarely been used in the field of palliative care [40, 53]. Studies comparing different indicators of spatial accessibility in this context are also unknown. To achieve a more precise assessment of spatial access, various indicators for operationalizing spatial access are used and compared in this study. Using the example of inpatient hospices as part of specialist palliative care in Germany, regional differences in spatial access will be identified and illustrated. Potential differences in the results caused by methodological characteristics will be discussed.

## Methods

### Data sources and data preparation

The identification of hospices, including their bed capacities, was initially based on ‘Directory for Hospice and Palliative Care’ (DHPC) [54], a freely accessible web-based self-disclosure database of the German Association for Palliative Medicine (DGP). This contains all kinds of inpatient and outpatient facilities for palliative care and hospice care, such as PCU, PCT, inpatient hospices, volunteer hospice teams, and other services for both adults and children available in Germany. For this study, inpatient hospices for adults were included (*n* = 252). Additional facilities (*n* = 43) and their bed capacities were identified using the lists of the regional associations of the DHPV and further research. All information on identified facilities was manually verified (as of April 2022). The hospice locations were then geocoded.

For the cartographic representation, freely available data on administrative boundaries from the Federal Agency for Cartography and Geodesy (BKG) for the year 2020, covering municipalities and districts, were used [55]. The data set also included population data (also from 2020). For analysis, it is necessary to use area-based population data as a point feature that is representative for the area (district or municipality) [12]. The BKG dataset contains a point data file, labeled “municipality centres”. It represents the location where most people in the municipality live, not the geometric centre, which is also frequently used [55]. The road data routing network used for all accessibility analyses was provided by WiGeoGIS in 2019.

### Setting

**Fig. 1:**
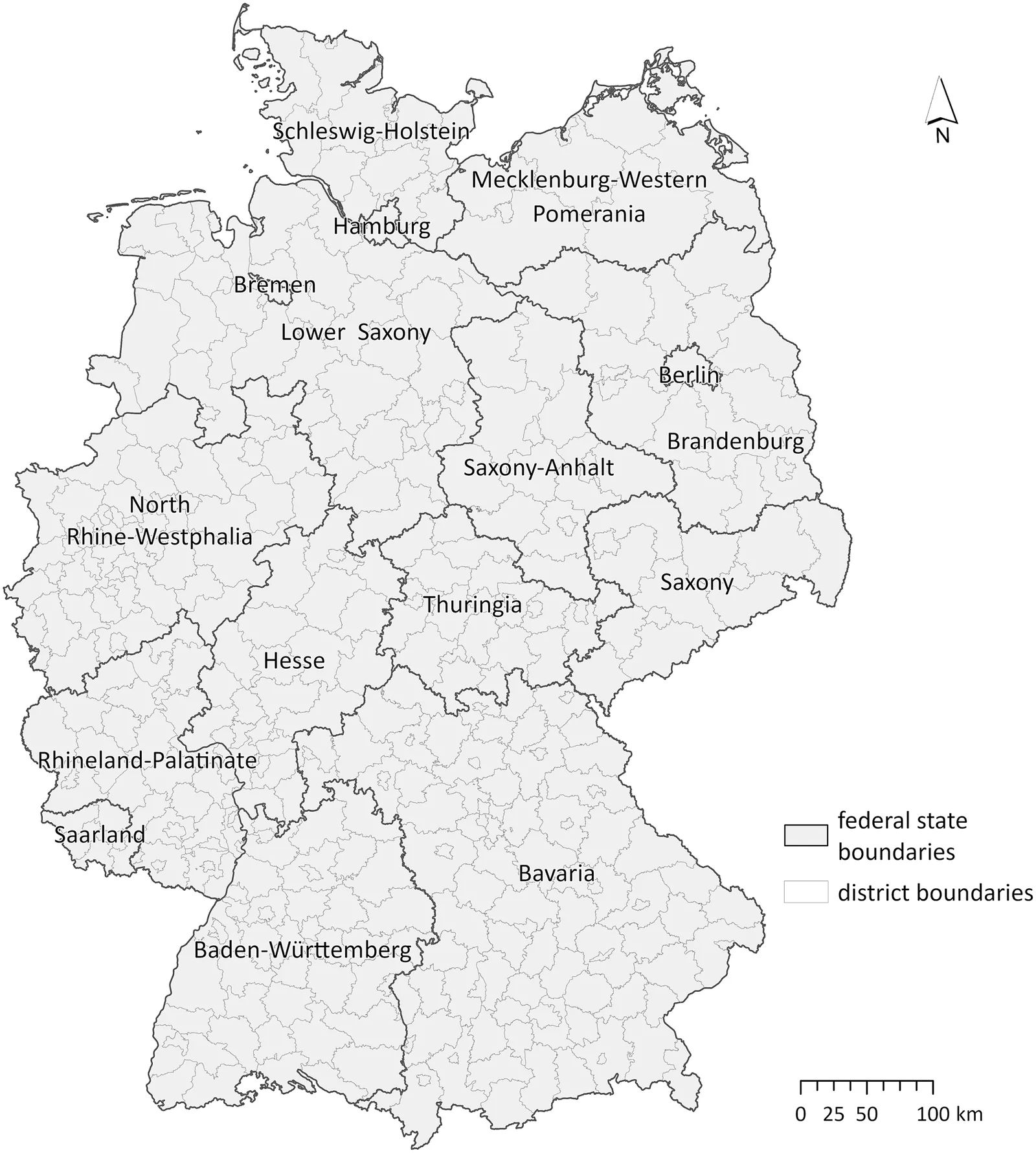
Overview of the study region in Germany

The study was conducted for the identified *n* = 295 inpatient hospices for adults in Germany which consists of 16 federal states, 401 districts and 10,995 municipalities. Figure 1 provides an overview of the study region. The total population in 2020 was 83,155,031 with a population density of 233 inhabitants per km^2^ [56]. Germany is characterized by regional differences in population density, age structure, and settlement patterns. It has numerous urban metropolitan areas that serve as centers of health care and provide services to the surrounding regions. At the same time, there are wide rural areas with a more elderly population, where access to health care services is often more limited. These geographic and demographic conditions influence access to health care services and can lead to regional disparities in care [57]. The planning and construction of inpatient hospices in Germany occurs within the framework of health self-governance, based on Section 39a of the German Social Code, Book V (SGB V), as well as agreements between health insurers and hospice providers. There is currently no centralised governmental planning of needs for inpatient hospices, as exists, for example, in the hospital sector. The assessment of needs is guided by recommendations from the European Association for Palliative Care (EAPC) and the DGP, as well as by empirical analyses that consider factors such as data on mortality, demographic factors, and existing care structures. The decision to establish new hospices is additionally influenced by initiatives from hospice providers, financial feasibility and regional funding structures [38, 58, 59].

## Data analysis

### Provider-to-population ratio

The PPR per 1 million inhabitants was calculated and visualized at district level by dividing the total number of beds in hospices by the total population. A more detailed visualization at municipal level was not used for this indicator due to a lack of significance. For better comparability of the results, the data was also aggregated at federal state level.

### Network-based travel time

Using the geocoded hospice locations, a network analysis was carried out and travel times by car as mode of transportation were calculated. This mode of transport was chosen as relatives and friends of patients in inpatient hospices, as well as general practitioners and physicians from PHC, use cars to drive to the hospices. Motorized private transport still represents the most common mode of transport in rural areas, where spatial access and public transport is assumed to be limited [60]. Patients, at the time of their admission, are transported there by ambulance services. By setting travel time thresholds, zones of equal potential accessibility are generated. In Germany, there are no official guidelines specifying how far away inpatient hospices or other palliative care services must be located. Based on other studies, thresholds of 15, 30 and 60 min were set [45, 49, 52]. In addition, the threshold of 30 min’ travel time is also mentioned in other contexts: for PCT in Germany, a document describes a maximum travel time of 30 min for care providers as desirable [61]. In Payne et al. (2022), it is noted that in inpatient hospices, care provided by a physician specialized in palliative care should be accessible within 30 min (and available 24 hours a day) [29]. There are no studies known to us on reasonable travel times for palliative care patients, so this information, as well as other studies, provide the only possible reference points. Service areas were linked to population data at the municipal level to identify the proportion of the population located within the catchment areas. In contrast to the provider-to-population ratios at the district level, the municipal level was chosen for this indicator to capture intra-district variability and enable a more fine-grained assessment of spatial accessibility by using smaller-scale data [12].

### FCA methods

Out of the FCA method family, the 2SFCA method [13] and the E2SFCA method [14] were applied, as they offer a balance of methodological transparency, empirical reproducibility and differentiated measurement of spatial accessibility. In particular, by integrating a distance decay function, the E2SFCA enables a more realistic representation of the decrease in accessibility with increasing distance without creating high complexity [15]. For both methods, the spatial accessibility index ($SPA{I_i}$) was calculated in a two-step process (see Table 1). First, a supply-demand ratio $PP{R_j}$was calculated for each inpatient hospice. Then, $SPA{I_i}$ was calculated for each population center at the municipal level.

**Table 1:** Calculation formula of the 2SFCA and E2SFCA method

	2SFCA	E2SFCA
Step 1: provider-to-population ratio ($PP{R_j}$)	$PP{R_j} = {{{S_j}} \over {\mathop \sum \nolimits_{i \in \left\{ {{d_{ij}} \le {d_{max}}} \right\}} {P_i}}}$	$PP{R_j} = {{{S_j}} \over {\mathop \sum \nolimits_{i \in \left\{ {{d_{ij}} \le {d_{max}}} \right\}} {P_i}{W_r}}}$
Step 2: spatial accessibility index ($SPA{I_i}$)	$SPA{I_i} = \mathop \sum \limits_{j \in \left\{ {{d_{ij}} \le {d_{max}}} \right\}} PP{R_j}$	$SPA{I_i} = \mathop \sum \limits_{j \in \left\{ {{d_{ij}} \le {d_{max}}} \right\}} PP{R_j}{W_r}$

${S_j}$ = supply capacity, ${P_i}$ = population at location *i*; ${d_{ij}}$ = distance between *i* and *j*; ${d_{max}}$ = maximum radius/maximum catchment size; ${W_r}$ = distance decay function/weight

**2SFCA:** To calculate the supply-demand ratio $PP{R_j}$ a maximum catchment area (d_max_), in this case within 30-min car travel time, was defined for each hospice *j* [45, 49, 52], and the catchment areas were calculated using network analysis. Then, all population centers *i* within the catchment areas (${d_{ij}}$≤d_max_) were identified and summed up $\sum{P_i}$. The sum was set in relation to the bed capacities of the inpatient hospices ${S_j}$ and a supply-demand ratio $PP{R_j}$ was formed.

In the second step, the $SPA{I_i}$ was created. For each population center *i*, a travel time radius of 30 min was calculated and then all provider-to-population ratios $PP{R_j}$ were summed up. Finally, the $SPA{I_i}$ was formed for each population centroid *i* at the municipal level. The $SPA{I_i}$ is higher the more beds there are in inpatient hospices, the faster the units can be reached, and the lower the population within a 30-min radius.

**E2SFCA:** E2SFCA method was applied in the same way as the 2SFCA. The maximum radius of 30 min’ travel time was additionally divided into three subzones (*r*_1,2,3_= 0–10, 10–20, 20–30 min). By defining a weighting ${W_r}$ for each subzone, a weighted supply-demand ratio was calculated. Similarly, the $PP{R_j}$ was summed up in the second step according to the weighting.

The distance decay function used is based on the original work by Luo and Qi (2009), which employs a Gaussian normal distribution. Two variants were used: a slow distance decay (1.0; 0.68 and 0.22) and sharp distance decay (1.0; 0.42 and 0.03) [14].

The results are presented as quintiles (Q1–Q5). Municipalities that do not have access to hospices within 30 min represent a separate additional category (“no access”). The category “no access” (zero values) was excluded from the quintile ranking to enable a differentiated analysis of the distribution of the accessibility index among the municipalities actually served. Based on literature [18], the quintiles were divided into a low (Q1 and Q2), medium (Q3), and high $SPA{I_i}$ (Q4 and Q5).

All analyses were conducted using ArcGIS Pro 2.9 (Esri Inc., California, Redlands, USA).

## Results

### Provider-to-population ratio

**Fig. 2:**
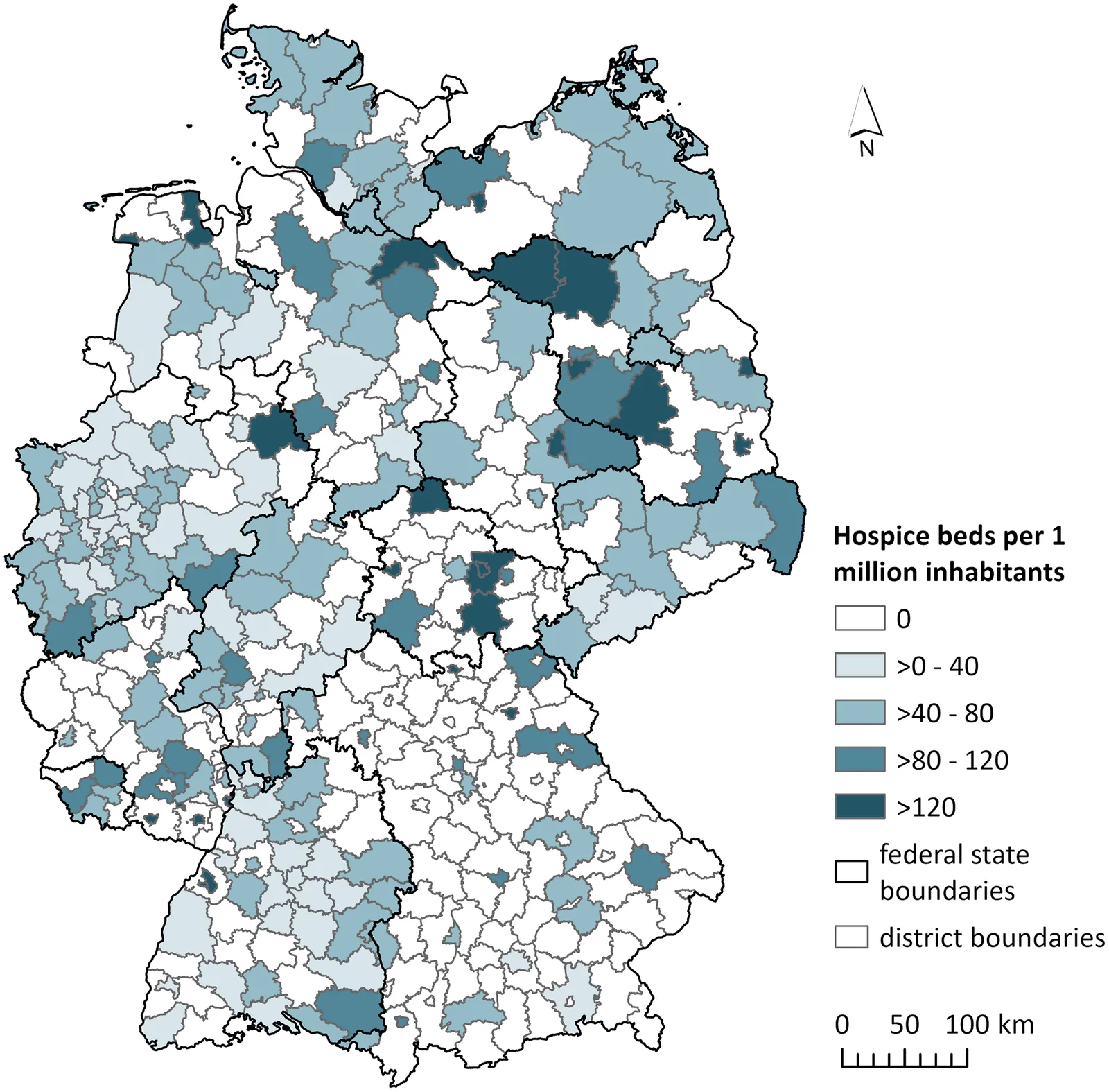
Provider to population ratio in Germany per 1,000,000 inhabitants on district level

A total of *N* = 295 hospices with a bed capacity of 3013 and an average capacity of 10 beds (range 2–16) were identified. 211 of the 401 districts have at least one inpatient hospice. This results in a ratio of 36.4 beds per 1 million inhabitants. Figure 2 shows the nationwide PPR at district level. Only the federal states of Berlin, Bremen and Hamburg (which are also city states) provide hospices in all districts. Bavaria has the fewest hospice beds with 16.8 beds per 1 million inhabitants, Brandenburg the most (64.8 beds per 1 million inhabitants; for further information, see Table 2). The districts with the most hospice beds per million inhabitants are Pirmasens in Rhineland-Palatinate (298.7), Eisenach in Thuringia (285.9) and Coburg in Bavaria (244.9). Apart from Berlin (*n* = 16), the districts of Hamburg (*n* = 8) and Lippe (*n* = 8) have the most hospice locations within a district.

**Table 2:** Hospice beds in inpatient hospices in Germany by federal state

Federal state	Hospice beds	Inhabitants	Beds per 1 million inhabitants
Baden-Württemberg	311	11,103,043	28.0
Bavaria	221	13,140,183	16.6
Berlin	229	3,664,088	62.5
Brandenburg	164	2,531,071	64.8
Bremen	24	680,130	35.3
Hamburg	106	1,852,478	57.2
Hesse	228	6,293,154	36.2
Mecklenburg-Vorpommern	90	1,610,774	55.9
Lower Saxony	285	8,003,421	35.6
North Rhine-Westphalia	716	17,925,570	39.9
Rhineland-Palatinate	127	4,098,391	31.0
Saarland	56	983,991	56.9
Saxony	164	4,056,941	40.4
Saxony-Anhalt	70	2,180,684	32.1
Schleswig-Holstein	124	2,910,875	42.6
Thuringia	98	2,120,237	46.2
**Germany**	**3013**	**83,155,031**	**36.4**

### Network-based travel time

**Fig. 3:**
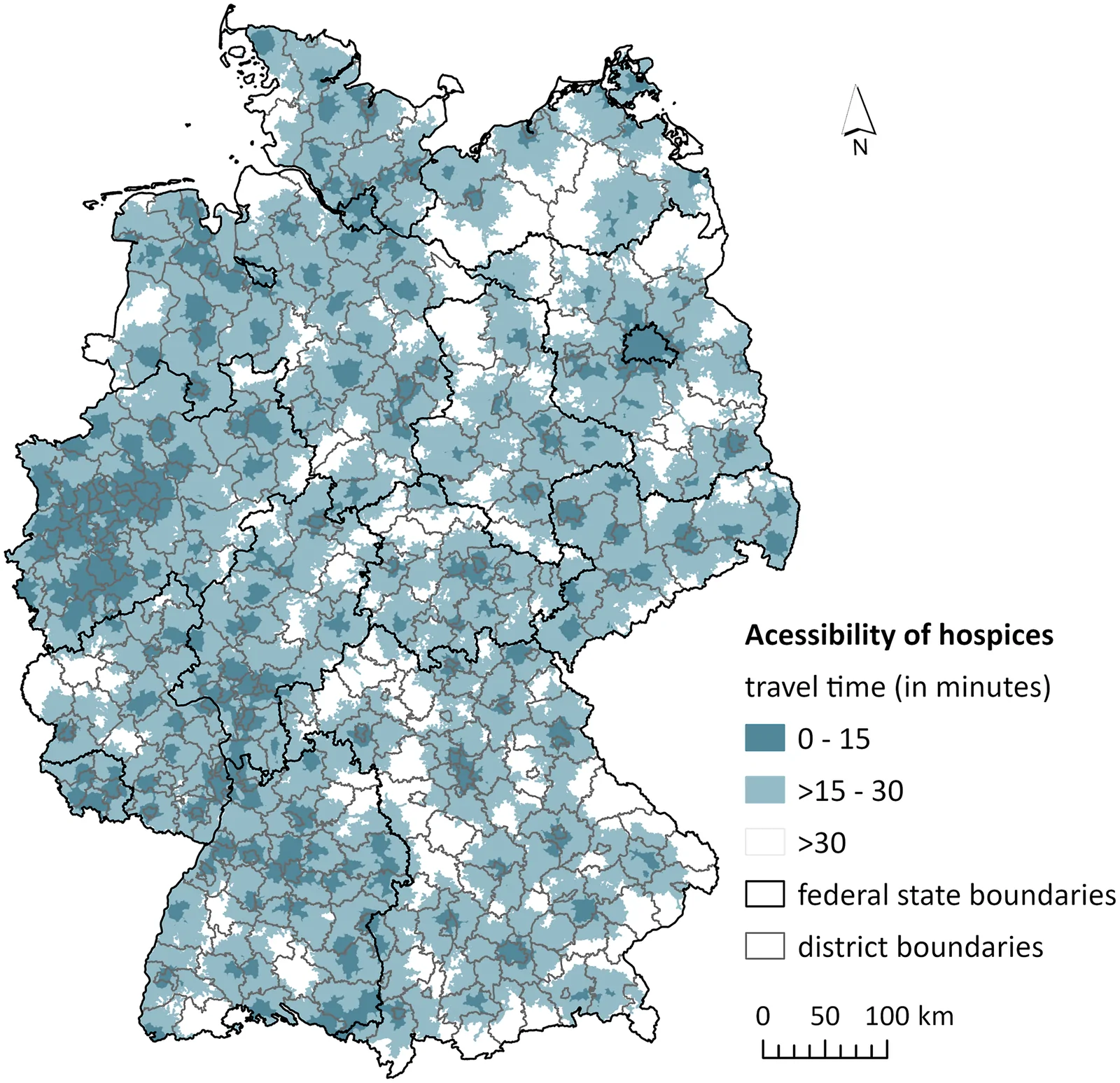
Travel time (in min) to inpatient hospices in Germany

The results of the Germany-wide network analysis shown in Fig. 3 indicate regional differences in the timely accessibility of hospices. Their location is particularly concentrated in high population centers (e.g., the Ruhr area or the Rhine-Main area) and larger cities. In more rural districts, hospices are often located in the district capitals (center effect). This effect is visible in many districts in Lower Saxony, for example. In Bavaria and Mecklenburg-Western Pomerania, as well as parts of Rhineland-Palatinate and Saxony-Anhalt, there are many contiguous areas where a hospice cannot be reached within a 30-min drive. Nevertheless, 58.8% of the population across Germany can reach a hospice within a 15-min drive by car, while 31.5% need between 15 and 30 min to get there. This means that a total of 90.3% can reach a hospice within a 30-min drive by car. 9.7% of the population need more than 30 min to drive to the nearest hospice. The absolute population figures are shown in Table 3.

**Table 3:** Percentage and total number of residents living in service areas of different driving times to inpatient hospices

Travel time (in min)	n	%
0 - 15	48,865,956	58.8
15 - 30	26,171,155	31.5
Subtotal 0 - 30	75,037,111	90.3
30 - 45	7,227,549	8.7
45 - 60	690,687	0.8
Subtotal 30 - 60	7,918,236	9.5
Subtotal > 60	199,684	0.2
**Total**	**83,155,031**	**100.0**

### FCA methods

**Fig. 4:**
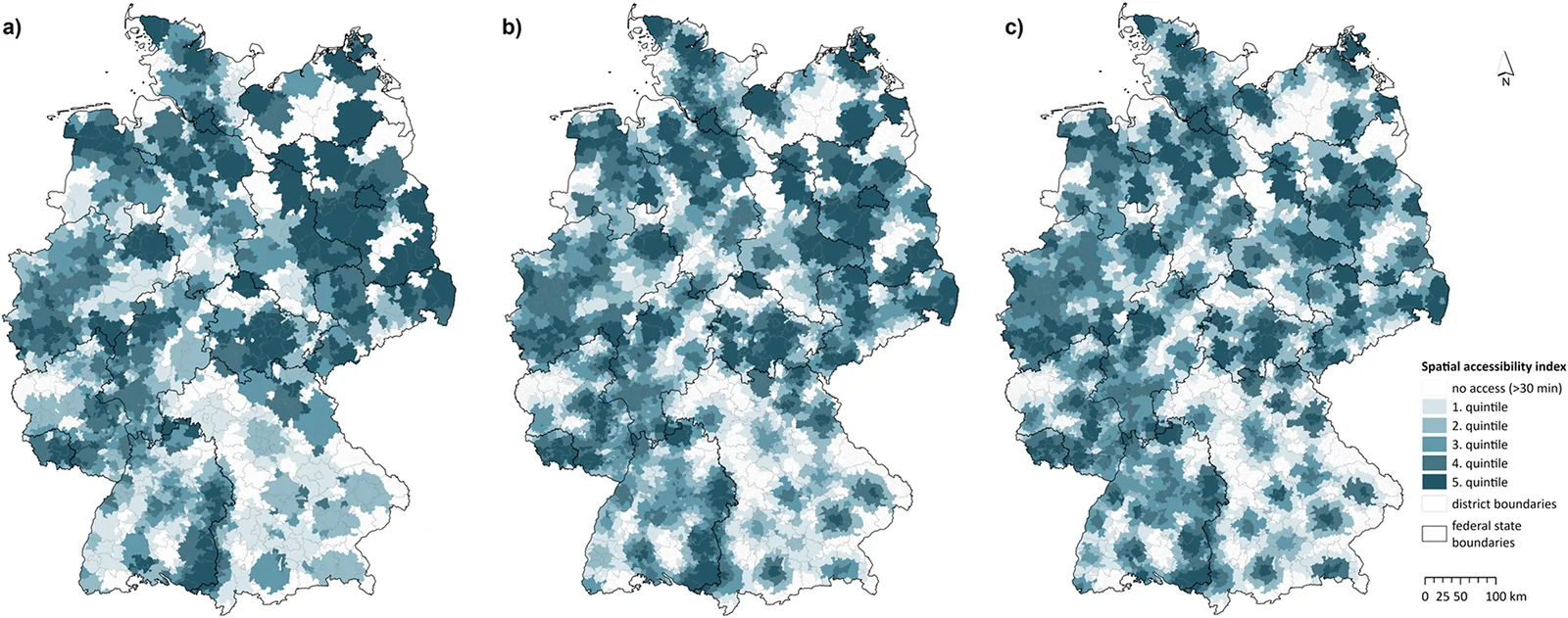
Spatial accessibility to hospices at municipal level in Germany. Spatial accessibility to inpatient hospices in Germany at the municipal level. Results of the floating catchment area methods are presented for **a**) 2SFCA, **b**) E2SFCA with slow distance decay, and **c**) E2SFCA with sharp distance decay. The $SPA{I_i}$ is categorized into quintiles: low (Q1, Q2), medium (Q3), and high (Q4, Q5)

The results obtained using FCA methods show a heterogeneous pattern. 2,138 of the 10,995 municipalities do not have access to inpatient hospices within a 30-min travel time (category: no access). This affects a total of 5,441,073 inhabitants, which corresponds to 6.5% of the population. Applying the 2SFCA method yields a very uniform $SPA{I_i}$ within municipalities located within a 30-min travel radius of inpatient hospices (see Fig. 4a). This results in sharp transitions between municipalities inside and outside the catchment area. In Bavaria in particular, the concentric circles can be defined as catchment areas around the hospice locations, as these are scattered across the entire state. One-third of Bavarian municipalities have no access to a hospice within 30 min, while 50.0% of municipalities have low accessibility (28.0% in Q1, 22.0% in Q2). In municipalities with an overlap of several catchment areas of hospice locations, a higher $SPA{I_i}$ can be observed. Another federal state with a large proportion of municipalities with low accessibility is Baden-Württemberg (Q1+Q2 = 46.0%). Federal states with a particularly high proportion of municipalities with high access indices are Brandenburg (71.0% in Q4 and Q5), Mecklenburg-Western Pomerania (51.0% in Q4 and Q5), Saarland (81.0% in Q4 and Q5), and Saxony (63.0% in Q4 and Q5). In addition, there are federal states in which $SPA{I_i}$ is distributed almost evenly across the quintiles (Schleswig-Holstein).

After applying the E2SFCA method, there are changes in the $SPA{I_i}$ (see Figs. 4b and 4c). The proportion of municipalities without access (within a 30-min drive) remains unchanged. The catchment areas of the hospices are graded in $SPA{I_i}$ according to the weightings applied. Municipalities in the immediate vicinity of the hospices often have high access indices. Peripheral municipalities tend to have low $SPA{I_i}$. The transitions to municipalities without access to areas outside the catchment areas appear smoother. This effect is most noticeable where the catchment areas overlap rarely (e.g., Bavaria). The E2SFCA method, particularly with a sharp distance decay function, underlines the geographical proximity to hospice locations. There is an increase in the proportion of municipalities with low access indices and a decrease in Q4 and Q5 in some federal states. This effect is seen particularly in Brandenburg, Saxony-Anhalt, and Thuringia (see Table 4). In Saxony, too, there is a shift toward medium values, although the proportion with high $SPA{I_i}$ remains high (slow distance decay function 48.0% in Q4 and Q5; sharp distance decay function 37.0% in Q4 and Q5). In Baden-Württemberg and Bavaria, in contrast, there is a shift from low towards medium $SPA{I_i}$.

**Table 4:** Proportion of municipalities by accessibility quintile (SPAIᵢ) by federal state

	2SFCA						E2SFCA slow distance decay						E2SFCA sharp distance decay					
	no SPA	Q1	Q2	Q3	Q4	Q5	no SPA	Q1	Q2	Q3	Q4	Q5	no SPA	Q1	Q2	Q3	Q4	Q5
BW	8%	27%	19%	22%	15%	10%	8%	18%	21%	27%	16%	10%	8%	16%	12%	33%	20%	11%
BY	29%	28%	22%	12%	6%	3%	29%	26%	20%	10%	11%	4%	29%	29%	12%	13%	13%	5%
BE	0%	0%	0%	0%	0%	100%	0%	0%	0%	0%	0%	100%	0%	0%	0%	0%	0%	100%
BB	24%	3%	1%	2%	16%	55%	24%	3%	9%	14%	15%	35%	24%	3%	24%	9%	12%	29%
HB	50%	0%	0%	50%	0%	0%	50%	0%	0%	50%	0%	0%	50%	0%	0%	0%	50%	0%
HH	0%	0%	0%	0%	0%	100%	0%	0%	0%	0%	0%	100%	0%	0%	0%	0%	0%	100%
HE	6%	8%	25%	23%	27%	12%	6%	13%	13%	28%	28%	12%	6%	12%	13%	28%	27%	14%
MV	39%	0%	0%	10%	18%	33%	39%	6%	10%	9%	8%	28%	39%	11%	13%	8%	11%	19%
NI	18%	17%	6%	23%	18%	18%	18%	12%	16%	16%	18%	20%	18%	10%	20%	12%	17%	22%
NW	3%	16%	13%	18%	29%	20%	3%	14%	12%	19%	35%	17%	3%	11%	10%	22%	34%	21%
RP	19%	13%	27%	16%	18%	7%	19%	18%	16%	18%	18%	12%	19%	17%	17%	17%	18%	13%
SL	0%	4%	4%	12%	42%	39%	0%	4%	6%	6%	31%	54%	0%	4%	6%	4%	46%	40%
SN	9%	5%	9%	15%	25%	38%	9%	7%	12%	24%	15%	33%	9%	9%	22%	23%	6%	31%
ST	26%	13%	13%	16%	9%	23%	26%	18%	22%	6%	12%	16%	26%	18%	19%	13%	10%	14%
SH	16%	14%	11%	19%	19%	21%	16%	11%	17%	16%	18%	22%	16%	9%	20%	12%	15%	29%
TH	16%	13%	14%	11%	14%	33%	16%	19%	12%	10%	14%	29%	16%	19%	20%	9%	13%	23%

Shown is the proportion of municipalities with low (Q1, Q2), medium (Q3), and high access (Q4, Q5) as well as no access within a 30-min travel time to inpatient hospices (no SPA), based on the Spatial Accessibility Index (SPAIᵢ)

BW = Baden-Württemberg; BY = Bavaria; BE = Berlin; BB = Brandenburg; HB = Bremen; HH = Hamburg; HE = Hesse; MV = Mecklenburg-Western Pomerania; NI = Lower Saxony; NW = North Rhine-Westphalia, RP = Rhineland-Palatinate; SL = Saarland; SN = Saxony; ST = Saxony-Anhalt; SH = Schleswig-Holstein; TH = Thuringia

## Discussion

### Provider-to-population ratio

The study is the first national evaluation of spatial accessibility to inpatient hospices in Germany, utilizing various indicators to operationalize spatial accessibility. One of the most applied indicators is the PPR. With 295 hospices and a national PPR of 36.2 beds/1 million inhabitants, availability has increased compared to earlier studies (27.3 beds/1 million inhabitants in 217 hospices in Melching (2016); 28.2 beds/1 million inhabitants in 209 hospices in Jansky et al. (2017); 25.6 beds/1 million inhabitants in 206 hospices in Prütz and Saß (2017) [24, 38, 46]. The pallCompare Monitor provides approximately similar figures, although the number of hospice beds recorded (2,754 hospice beds) is lower than the figure found in this study (3,013 beds) [47]. Since all earlier studies mentioned refer solely to the DHPC as a database, which did not include all hospice locations, this may have led to an underestimation of availability. The EAPC’s reference value of 40–50 beds/1 million inhabitants refers to both beds in inpatient hospices and PCU and is therefore not comparable [29]. A recent study by the EAPC estimated 1.13 specialist palliative care services per 100,000 inhabitants in Germany, which is above the median in a European comparison (0.96 services per 100,000 inhabitants), but only in the second quintile [23]. Due to our focus on inpatient hospices in this study, it is difficult to compare the results directly. “Geographic reach”, as an indicator for the provision of specialist palliative care services used in the EAPC Atlas of Palliative Care in the European Region 2025 is rated at the highest level (integrated) for Germany. However, the ratings of “Geographic reach” are based on the assessment of the experts consulted, and measurable accessibility as a spatial component was not included [23]. Overall, the existing guidelines and recommendations from the DGP and EAPC are based solely on ratios [29]. This kind of indicator is easy to understand and interpret and may provide a starting point to analyze the healthcare provision situation [62]. However, its simplicity limits its validity and does not adequately reflect the healthcare provision situation. Cross-border relationships between neighbouring regions are not taken into account, even though it is well known that people’s mobility is not restricted by administrative boundaries [7, 18, 63]. In addition, the supply is dichotomized. A hospice is only considered accessible if it is located within the administrative unit. Furthermore, it is considered equally accessible regardless of where someone is located within the defined area [18, 64–66]. If the districts cover a very large area, this can result in an overestimation of hospice availability (e.g., rural areas like Mecklenburg-Western Pomerania or Brandenburg). If, on the other hand, a hospice is located in an urban district (cities which constitute a district in their own right), the surrounding area may appear to have low availability, even though a contributory effect can be assumed (e.g. Bavaria). Choosing the analytical unit therefore implies a certain degree of arbitrariness (Modifiable Area Unit Problem, MAUP) [18, 64, 67].

### Network-based travel time

Network-based travel time analysis with GIS, combined with population data, is a frequently used indicator to assess potential accessibility of care facilities. In this study, it was applied for the first time to inpatient hospice care throughout Germany. A large proportion of inhabitants (90.3%) can reach inpatient hospices within 30 min by motorized individual transport. Gesell et al. (2023), who examined the accessibility of specialist palliative care services (PCU and PCT) in Germany, reported similarly high results for PCU (86.0%) and PCT (87.2%) [45]. In Switzerland (95.0%), Ireland (84.0%) and Spain (79.0%), large proportions of the population also reach specialist palliative care services within 30 min’ travel time by private motorized transport, although it is not entirely clear whether inpatient hospices were included in this study [52]. By taking into account the transport infrastructure and topographical conditions, network analyses are generally more valid than the use of Euclidean distance (as the crow flies) as in Wiese et al. (2010) [48]. However, this indicator also has some limitations. Information on availability and demand competition is not taken into account. In addition, the restriction to motorized private transport leads to a bias [7, 18, 63]. In Germany, 77.3% of private households have at least one car and car-sharing services are increasingly available [68]. However, general mobility decreases with increasing age. People over the age of 80 in particular are dependent on alternative mode of transportation (public transport, on-call buses, shared cabs) [18, 60, 69]. In addition, the perception of distance changes among older people, and their willingness to travel further distances decreases [70]. Relying on public transport can aggravate the situation, especially in rural areas [71, 72]. There is no nationwide study in the hospice and palliative care context, but Weinhold et al. (2022) estimate that 21% of the population in Saxony can reach inpatient hospices by public transport in 30 min and 51.0% in 60 min [40].

### FCA methods

By taking availability and accessibility into account, the 2SFCA is an important basic method for adequately mapping spatial accessibility [13]. We applied the 2SFCA and E2SFCA methods to inpatient hospices in Germany for the first time, providing a more complex methodological approach to investigate spatial accessibility. Previously, studies have only been carried out at federal state level in Saxony, which showed quite similar results regarding the distribution pattern of the $SPA{I_i}$ [40]. In terms of accessibility, using flexible catchment areas based on hospice and population locations is a step forward compared to the orientation towards fixed administrative boundaries. This allows the mobility behavior of the population (cross-border service-seeking behavior) to be mapped more realistically. This is particularly relevant for regions close to the border, such as between the federal states of Baden-Württemberg and Bavaria, where there is a clustering of hospice locations and high accessibility indices. At the same time, demand competition can be taken into account by considering the overlap of catchment areas and availability of hospice beds. The data used as a demand population is also relevant. The general population was used here as we considered both patients and relatives as a demand population. Other studies used the entire deceased population [40] or deceased cancer patients as a potential demand population [49]. Another limitation of the 2SFCA is the dichotomous approach to catchment areas, in other words the assumption that all areas are considered equally accessible, which generates hard transitions in the $SPA{I_i}$ between communities inside and outside the catchment area. In addition, the $SPA{I_i}$ in the periphery of a catchment area is overestimated due to overlapping catchment areas [14, 18]. Further, excluding zero values (=category ‘no access’) from quintile classification resulted in a more differentiated distribution of the accessibility index within the municipalities with access. It improved the distinction between low but existing levels of accessibility, as the quintile thresholds were not shifted towards lower values by the high number of municipalities with no access.

Using E2SFCA, a differentiation within the catchment areas is achieved by applying a distance decay function. A concentric pattern is created, with high access indices near the hospice locations and lower values in peripheral areas. This pattern is particularly prominent using sharp distance decay function. Slow distance decay function causes a smoothing effect. Compared to 2SFCA, it is an important step towards operationalizing spatial access more realistically [14, 18, 65, 66]. In areas with low density of inpatient hospices, application of E2SFCA causes more changes in the $SPA{I_i}$ compared to 2SFCA. High accessibility indices occur in proximity to inpatient hospices. In areas where many hospices are clustered, accessibility indices change less (e.g. Saarland, Ruhr area, city states) compared to areas with a lower density of hospices. These are mostly urban areas where the population density is high and the supply-demand situation is complex. In a similar study on PCU, we linked the municipalities with a city and municipality classification of the Federal Institute for Research on Building, Urban Affairs, and Spatial Development (BBSR) [53]. The proportion of municipalities without access within 30 min in rural municipalities was higher than in urban municipalities, while access indices in urban areas tended to be higher overall [53]. A lower availability of hospice and palliative care services in low-population areas with an older population is also described in other German [59] and international studies [49, 73]. Finally, it is open to debate which form of distance decay function is most suitable for hospice and palliative care when using E2SFCA method. Similar arguments to those used in discussing maximum travel time radius may be applied to this question. On the one hand, it is a highly specialist care area that is only used by a small group of people, similar to highly specialist oncological or other care, which suggests a slow distance decay function [14]. On the other hand, as previously discussed, end-of-life care is a particularly sensitive area where long travel times are less acceptable for patients and relatives. Overall, the results of the FCA methods are less intuitive to interpret, but the methodology is considered as most suitable for operationalizing spatial access to healthcare services [65, 74]. Many studies use the methodology for descriptive purposes only, but some also use it as a potential explanatory variable for health purposes [15].

Consequently, the definition of a travel time of 30 min is a critical parameter that needs to be discussed. This applies to both network-based travel time and FCA methods, as network-based travel time forms the methodical basis for operationalizing travel time or accessibility in FCA methods [15]. There are no regulatory requirements for the maximum size of catchment areas or valid data on actual catchment areas of hospices in Germany. Other German studies used 30 min and 60 min as the maximum travel time to care providers of specialist palliative care [40, 45]. In Great Britain, which is very similar to the German distance dimensions, a maximum of 30 min was also considered acceptable [49], whereas in spacious Canada 60 min were considered appropriate [51]. Hospice and palliative care is a particularly sensitive area of healthcare where care should be provided close to home or the familiar living environment [75, 76]. Long transportation routes can prevent unstable, vulnerable and burdened patients from being transferred to a hospice. Frequent visits by relatives in the last period of life can support patients emotionally, psychologically and existentially. Relatives are often involved in the decision-making process regarding changes to treatment goals as proxy caregivers and are crucial for successful symptom relief [77]. However, relatives themselves may experience high stress levels [78], resulting in long travel times to be challenging. Emotions such as sadness, anger, stress, anxiety, fatigue and overall grief can have a negative impact on the ability to drive [79–81]. However, it was argued that travel times of 60 min to inpatient hospices can be considered just as justified as to other specialist facilities [63, 82].

#### Limitations and outlook

As already discussed, the study limited travel time to a maximum of 30 min. A sensitivity analysis with an extension of maximum travel time could provide additional information. An extension of the analysis to consider public transportation would also be useful. Complete coverage of all inpatient hospices cannot be guaranteed. It has been shown that entries in the DHPC, as a form of web-based self-reporting database, may be incomplete and may contain biases or inconsistencies in how facilities report their services. A limiting factor is the assumption that patients potentially use the hospice closest to their place of residence, and that their relatives live in the immediate neighborhood. Due to socio-demographic and social developments as well as globalization and migration processes, grown-up children are living at greater distances to their ageing parents. Long-distance caregiving is therefore becoming increasingly important [83], and patients may chose an inpatient hospice closer to their relatives place residence, instead of their own. Furthermore, a maximum travel time of 30 min may also be relevant for friends, neighbors and other important people from the patients’ original neighborhood.

In addition, using different aggregation levels of administrative areas or population data leads to inequalities in the presentation. In case of PPR, a calculation and presentation at municipality level would be too small-scale. Conversely, using population data at district level would be too large-scale for FCA and network-based travel time methods, which require smaller-scale population data, e.g. at grid cell level (1 km or 100 m grid) to yield more precise results. At the time of conducting the analysis, the 2022 census data at grid cell level was not yet available. This would also have allowed including further socio-demographic characteristics, such as the proportion of over 65-year-olds in the population, and thus a more precise mapping of potential hospice patients. As such data was not available at municipal level at the time of analysis, the overall population was used, which can lead to biases in view of regional differences in age structure.

Only a selection of indicators for operationalizing spatial access were presented and applied in this article. The 2SFCA method and E2SFCA method are two out of numerous, increasingly complex FCA methods. Meanwhile, there are many other methods that include numerous additional parameters and are able to model an even more realistic view of supply situations. However, these models are more complex and require an extensive presentation and discussion of methods that, in the end, will only be useful for very detailed use, but not for general consideration of stakeholders in the healthcare system [17, 18].

Overall, the study of potential spatial accessibility to inpatient hospices is based on theoretical considerations, for example, the number of beds indicates only theoretical availability [18, 74, 84]. Observations from practice have shown that there are often long waiting lists in hospices in Germany and the demand for beds is high [38, 40, 85]. A Canadian study integrated waiting times when using the 2SFCA [86]. Further, for a more realistic mapping of care situations, an intersectoral analysis of all existing palliative and hospice care structures in Germany should be conducted. Compared to other patient groups, it is not very useful to focus on individual groups of care structures, as the treatment pathways are very complex and cross-professional. In addition, taking into account the actual occupancy situation, possible capacity restrictions due to e.g. staff shortages, or patient waiting times due to high utilization, demographic characteristics (e.g. high regional average age, proportion of people with a migration history), socio-economic factors (e.g. proportion of single households) and utilization data can help to identify areas with low access and identify gaps in care. Quantitative and qualitative surveys of patients on realized travel times and their reasonability could provide valuable information on patient preferences. A complex (cross-sectoral) access analysis, taking into account other dimensions of access, can contribute to an improvement in the planning of hospice and palliative care close to home.

## Conclusion

This study examined the spatial accessibility of inpatient hospices in Germany using different indicators. The results show high spatial accessibility, but also illustrate that the chosen method has a considerable influence on the results. As the planning of new inpatient hospice locations in Germany is not governed by central authorities, it is not possible to address one specific stakeholders with policy recommendations. Decisions are largely determined at local level and therefore tend to be oriented towards district boundaries. By visualizing regions with no or poor access within a 30-min travel time, the study may encourage a more detailed examination of these areas to determine whether there are real gaps in inpatient hospice care provision in these communities. Although indicators of availability (provider-to-population ratios) or accessibility (network-based travel time) have the advantage of simplicity and reproducibility, relying solely on these methods would neglect an essential aspect and result in an incomplete assessment of spatial accessibility. The paper can raise awareness to consider advanced methods such as FCA methods when examining spatial accessibility to healthcare services, as these take both dimensions of accessibility equally into account. Although the E2SFCA method is more complex, we prefer to use this methodology as it is important to take the aspect of distance decay into account. As this is the first study to our knowledge in the palliative care context, both decay variants were included in the calculations to provide a platform for further discussion and research. Methods of health geography add great value and contribute to an improvement in the planning of palliative care services. Considering demographic change and increasing demand for hospice and palliative care, this research perspective is highly relevant.

## Data Availability

Data can be obtained from the corresponding author on reasonable request. Data on geographic information are freely available from the Service Center of the Federal Agency for Cartography and Geodesy at: (https:/gdz.bkg.bund.de/index.php/default/open-data.html).

## Abbreviations

2SFCA: Two-Step floating catchment area
BKG: Federal Agency for Cartography and Geodesy
BBSR: Federal Institute for Research on Building, Urban Affairs, and Spatial Development
DGP: German Association for Palliative Medicine
DHPC: Directory for Hospice and Palliative Care
DHPV: German Hospice and Palliative Care Association
E2SFCA: Enhanced Two-Step floating catchment area
EAPC: European Association for Palliative Care
FCA: Floating catchment area
MAUP: Modifiable Area Unit Problem
PPR: provider-to-population ratio
PCT: home palliative care team
PCU: Palliative care unit
SPAI_i_: Spatial accessibility index
